# Development of a food-based diet quality score for Japanese: associations of the score with nutrient intakes in young, middle-aged and older Japanese women

**DOI:** 10.1017/jns.2016.36

**Published:** 2016-10-28

**Authors:** Nozomi Kuriyama, Kentaro Murakami, M. Barbara E. Livingstone, Hitomi Okubo, Satomi Kobayashi, Hitomi Suga, Satoshi Sasaki, the Three-generation Study of Women on Diets and Health Study Group

**Affiliations:** 1 Department of Nutrition, School of Human Cultures, University of Shiga Prefecture, Shiga, Japan; 2 Northern Ireland Centre for Food and Health, Ulster University, Coleraine, UK; 3 Department of Health Promotion, National Institute of Public Health, Saitama, Japan; 4 Department of Social and Preventive Epidemiology, School of Public Health, University of Tokyo, Tokyo, Japan; 5 Department of Social and Preventive Epidemiology, Graduate School of Medicine, University of Tokyo, Tokyo, Japan

**Keywords:** Diet quality, Diet score, Women, Japan, BDHQ, brief-type diet history questionnaire, DHQ, diet history questionnaire, EI, energy intake, PAL, physical activity level

## Abstract

Several previous studies have shown that a diet score based on the Japanese food guide Spinning Top (the original score) is associated with both favourable and unfavourable dietary intake patterns. We developed a food-based diet quality score (the modified score) and examined associations with nutrient intakes. Subjects were 3963 young (all aged 18 years), 3800 middle-aged (mean age 47·7 (sd 3·9) years) and 2211 older (mean age 74·4 (sd 5·2) years) Japanese women. Dietary intakes were assessed using comprehensive (for the young and middle-aged) and brief-type (for the older) diet history questionnaires. The original score was calculated based on intakes of grains, vegetables, fish/meat, milk, fruits, and snacks/alcoholic beverages. The modified score was similarly calculated, but included Na from seasonings and without applying the upper cut-off values for dietary components where increased consumption is advocated for Japanese women (grains, vegetables, fish/meat, milk, and fruits). The original score was positively associated with intakes of carbohydrate, dietary fibre, and all the vitamins and minerals examined including Na and inversely with intakes of fats and alcohol in young and middle-aged women. In older women, the original score was inversely associated with intakes of all nutrients except for carbohydrate and vitamin C. However, the modified score was associated positively with intakes of protein, carbohydrate, dietary fibre, K, Ca, Mg, Fe, vitamins A, C and E, and folate and inversely with intakes of fats, alcohol and Na in all generations. In conclusion, the modified diet score was positively associated with favourable nutrient intake patterns in Japanese women.

Traditionally, nutritional research has concentrated on the detailed examination of possible health roles and consequences of dietary components (foods, nutrients or both) considered in isolation. Nevertheless, the effects of individual foods and nutrients on health are usually difficult to estimate, because they can be small^(^^1^^)^. Furthermore, nutrients and foods are consumed in combination and their combined effects may be interactive or synergistic^(^^2^^)^. Consequently, nutritional research has expanded to consider the whole diet in addition to the individual components.

Traditional dietary cultures and patterns of the Japanese have long been of interest worldwide because of, for example, the low prevalence of CHD and the long life expectancy in Japan^(^^3^^–^^5^^)^. The Japanese dietary pattern has several characteristics seldom observed in those of Western populations, including high intakes of rice, soyabean products, fish, seaweeds and green tea and low intakes of animal fat and soft drinks^(^^6^^)^. The Japanese food guide Spinning Top was developed in 2005 by the Japanese Ministry of Health, Labour and Welfare and the Ministry of Agriculture, Forestry and Fisheries, based on the food-based Dietary Guidelines for Japanese, which was formulated in 2000 by the former Ministry of Education, the former Ministry of Health and Welfare, and the Ministry of Agriculture, Forestry and Fisheries^(^^7^^)^.

A limited number of studies have assessed the adherence to the food-based Japanese dietary guidelines on the basis of the Japanese food guide Spinning Top, which consists of the following food categories: grain dishes (rice, bread, noodles, etc.); vegetable dishes (vegetables, mushrooms, potatoes and seaweed); fish and meat dishes (meat, fish, eggs, soyabeans, etc.); milk (milk and milk products); fruits; snacks and alcoholic beverages. Higher adherence to the dietary guidelines was not only prospectively associated with lower future mortality in middle-aged women (but not in men)^(^^8^^)^ but also cross-sectionally associated with lower waist circumference and LDL-cholesterol concentrations in young women^(^^9^^)^. However, it was also associated with both favourable (e.g. higher intakes of dietary fibre^(^^8^^,^^9^^)^ and micronutrients^(^^8^^,^^9^^)^) and unfavourable (e.g. higher intakes of saturated fat^(^^8^^)^ and Na^(^^8^^,^^9^^)^) aspects of dietary quality. A major problem of this score is that Na was excluded even though high intakes are a serious concern in Japanese diets^(^^10^^–^^12^^)^. The major food group contributing to Na intake is seasonings such as salt, miso and soya sauce^(^^13^^)^. Additionally, Japanese women are characterised by an increasing prevalence of underweight (as well as no increase of prevalence of obesity)^(^^6^^,^^14^^,^^15^^)^ and lower intakes of many micronutrients in addition to lower-fat intakes^(^^6^^,^^16^^,^^17^^)^. Considering this, another problem is the use of upper cut-off values for the maximum score for dietary components where higher intakes are advocated at least for Japanese women (i.e. grain dishes; vegetable dishes; fish and meat dishes; milk; and fruits). Given the difficulty or uncertainty in determining the maximum amount of these components, one solution is to employ least-restrictive standards; those that are easiest to achieve among recommendations^(^^18^^)^. A modified diet score taking these problems into account would be associated with nutrient intakes in the expected directions only.

The aim of the present cross-sectional study in young, middle-aged and older Japanese women was to calculate two diet scores based on the original and modified scoring systems and to examine the associations with nutrient intakes. The original score was calculated based on the Japanese food guide Spinning Top, in accordance with the procedure used in previous studies^(^^9^^)^, while the modified score took into account Na intake from seasonings and did not apply the upper cut-off values for those dietary components where increased consumption is recommended.

## Subjects and methods

### Survey design

The present cross-sectional study was based on data from the Three-generation Study of Women on Diets and Health, a self-administered questionnaire survey conducted in northern and western Japan in 2011 and in eastern Japan in 2012. Details of the study design and survey procedure have been published elsewhere^(^^19^^)^. Briefly, a total of 7016 dietetic students from eighty-five higher education institutions in thirty-five of forty-seven prefectures in Japan were asked to complete two questionnaires on dietary habits and lifestyle factors which were distributed during the orientation session or the first lecture designed for freshmen in April 2011 or 2012. Each student was also requested to directly distribute similar questionnaires to his or her mother and grandmother and invite them to join the study. Recruitment priority for the grandmother generation was given to the maternal, or, if unavailable, paternal grandmother, followed by his or her female acquaintance aged 65–89 years. In total, 4933 students, including 4656 women and 277 men (response rate: 70·3 %), 4044 mothers (57·6 %), and 2332 women from the grandmother generation (33·2 %) answered both questionnaires.

The present study was conducted according to the guidelines laid down in the Declaration of Helsinki and all procedures involving human subjects were approved by the Ethics Committee of the University of Tokyo Faculty of Medicine. Written informed consent was obtained from each subject and also from a parent or guardian for subjects aged <20 years.

### Analytic sample

In the present study, we considered that the students (excluding males), mothers and grandmothers (including acquaintances) consisted of groups of young, middle-aged and older women, respectively.

For the analysis of young women, we selected female students aged 18 years (*n* 4065). We then excluded those living in eastern Japan who participated in the 2011 survey, given the influence of the Great East Japan Earthquake in March 2011 (*n* 39), those who answered the questionnaires after 19 May to minimise the influence of dietetic education (*n* 56), and those whose data were derived from the institution where the response rate was extremely low (2 %) (*n* 2). We further excluded those with missing information on the variables of interest (*n* 5).

For the analysis of middle-aged women, we selected mothers aged ≤60 years (*n* 4012). We then excluded those living in eastern Japan who participated in the 2011 survey (*n* 63), and those from the institution with the low response rate (*n* 2). We further excluded those with erroneous or missing information on the variables of interest (*n* 147; mainly missing information on education).

For the analysis of older women, we selected grandmothers (and acquaintances) aged ≥61 years (*n* 2325). We then excluded those living in eastern Japan who participated in the 2011 survey (*n* 47), and those from the institution with the low response rate (*n* 1). We further excluded those with erroneous or missing information on the variables of interest (*n* 66). Consequently, the final sample sizes were 3963, 3800 and 2211 for young, middle-aged and older women, respectively.

### Dietary assessment

Dietary habits during the preceding month were self-reported using a comprehensive diet history questionnaire (DHQ) for young and middle-aged women^(^^20^^–^^22^^)^ and a brief-type diet history questionnaire (BDHQ) for older women^(^^20^^,^^21^^)^. Responses to the DHQ and the BDHQ, as well as to the lifestyle questionnaire, were checked at least once by survey staff at the study centre. If any missing or erroneous responses were given to questions which were considered essential for the analysis (including all the questions in the DHQ and BDHQ), the subject was asked to complete those questions again. Details of the structure and calculation method of dietary intake of the DHQ and BDHQ have been published elsewhere^(^^20^^–^^22^^)^. Briefly, the DHQ and BDHQ are structured questionnaires about the consumption frequency (and portion size in the DHQ) of selected foods commonly consumed in Japan, as well as general dietary behaviour and usual cooking methods^(^^22^^,^^23^^)^. Estimates of the daily intake of foods (151 items in the DHQ and fifty-eight items in the BDHQ), energy and selected nutrients were calculated using an *ad hoc* computer algorithm for the DHQ and BDHQ, which was based on the Standard Tables of Food Composition in Japan^(^^24^^)^. A relative validity of the DHQ and BDHQ has been previously investigated among ninety-two women aged 31–69 years using a 16-d dietary record as reference. Briefly, for the DHQ, the median value of Spearman's correlation coefficients of food groups was 0·43 (range: −0·09 to 0·77), and that of Pearson's correlation coefficients for the nutrients used in the present study was 0·57 (range: 0·39–0·87). The corresponding values for the BDHQ were 0·44 (range: 0·14–0·82) and 0·58 (range: 0·34–0·87), respectively^(^^20^^,^^21^^)^.

### Calculation of the Japanese diet scores

We calculated the original and modified Japanese diet scores based on the Japanese food guide Spinning Top. For the former (the original score)^(^^9^^)^, the following six categories were considered: grain dishes (rice, bread, noodles, etc.); vegetable dishes (vegetables, mushrooms, potatoes and seaweed); fish and meat dishes (meat, fish, egg, soyabeans, etc.); milk (milk and milk products); fruits; and snacks and alcoholic beverages. These have the recommended number of servings (for grain, vegetable, fish and meat dishes, milk, and fruits) or of total energy (for snacks and alcoholic beverages), depending on sex, age and physical activity (we assumed a low level of physical activity for all women because of the apparently predominantly sedentary lifestyle of our subjects as described below): grain dishes (4–5 servings/7531 kJ); vegetable dishes (5–6 servings/7531 kJ); fish and meat dishes (3–4 servings/7531 kJ); milk (2 servings/7531 kJ); fruits (2 servings/7531 kJ); snacks and alcoholic beverages (≤837/7531 kJ). By definition, one serving of grain dishes provided 40 g carbohydrate; one serving of vegetable dishes was 70 g; one serving of fish and meat dishes provided 6 g protein; one serving of milk provided 100 mg Ca; one serving of fruits was 100 g.

Using information on intakes of foods and energy derived from the DHQ or BDHQ, we calculated servings of grain dishes, vegetable dishes, fish and meat dishes, milk and fruits, and energy intake (EI) from snacks and alcoholic beverages, as described in Supplementary Table S1. These values were energy adjusted using the density method to obtain the values per 7531 kJ of energy to enable comparison with the recommended values. A participant who consumed the recommended number (of servings or energy) for each of the six categories received a score of 10 for that category (see Supplementary Table S1). For a participant who exceeded or fell short of the recommended number of servings (for grain, vegetable, fish and meat dishes, milk, and fruits) or exceeded the recommended amount of energy (for snacks and alcoholic beverages), the score was calculated proportionately between 0 and 10 (see Supplementary Table S1). For example, if a participant consumed two of the recommended five to six servings of vegetable dishes, the score was calculated as (10 × 2/5 = 4). Likewise, if a participant consumed six servings of fish and meat dishes when the recommendation was three to four servings, the score was (10–10 × (6–4)/4 = 5). When the calculation produced a negative score because of excess servings or energy, the score was converted to 0. The six scores were then summed to provide the overall score on adherence to the food-based Japanese dietary guidelines (the original score), which ranged from 0 to 60.

For the latter (the modified score), we considered seasonings in addition to the categories used in the original score. This was based on a serious concern about high Na intake in Japanese (mean: 5506 mg/d)^(^^12^^)^, more than half of which is derived from seasonings^(^^13^^)^. We used the 10th percentile of energy-adjusted Na intake from seasonings in older women (i.e. 1389 mg/7531 kJ) as a cut-off point. A participant who fell short of this value received a score of 10 for this category. For a participant who exceeded this value, the score was calculated proportionately. When the calculation produced a negative score because of excess Na intake, the score was converted to 0. For the other five categories (except for snacks and alcoholic beverages), the score was calculated similarly in the case of the original score, without using the upper cut-off values for the maximum score. In other words, we employed least-restrictive standards; those that are easiest to achieve among recommendations^(^^18^^)^. This decision was based on the difficulty or uncertainty in determining the maximum amount of dietary components where increased intakes are advocated for Japanese women. Given an increasing prevalence of underweight (as well as no increase of prevalence of obesity)^(^^6^^,^^14^^,^^15^^)^ as well as lower intakes of many micronutrients in addition to lower fat intakes among Japanese women^(^^6^^,^^16^^,^^17^^)^, we considered that increasing intakes of not only vegetables, fruits and fish but also grains, meat and dairy products may be helpful to improve overall diet quality. Thus, a participant who exceeded the lower recommendation value received a score of 10 for that category. The score for the snack and alcoholic beverages was calculated similarly to the case of the original score. The seven scores were then summed to provide the modified diet score, which ranged from 0 to 70.

### Assessment of other variables

All the variables used were based on the participants' self-reported information. Age at the time of the survey was calculated based on birth date. Residential area was grouped into six regions (Hokkaido and Tohoku, Kanto, Hokuriku and Tokai, Kinki, Chugoku and Shikoku, or Kyushu) and also into three categories according to population size (city with a population ≥1 million, city with a population <1 million, or town and village). Living status (not considered in middle-aged women because almost all lived with family) was grouped into three categories (living alone, living with family, or living with others), but for older women those living with others were added to those living with family because of the very small number of subjects (*n* 4). BMI was calculated as body weight (kg) divided by square of body height (m). Weight status was grouped into three categories: underweight (BMI <18·5 kg/m^2^), normal weight (BMI ≥18·5 to <25 kg/m^2^) and overweight (BMI ≥25·0 kg/m^2^)^(^^25^^)^. Information on current smoking, current alcohol drinking, dietary supplement use and medicine use (all yes or no) was also used. Eating out was categorised as ≤3 times/month, once per week, 2–3 times/week, or ≥4 times/week (not available in older women). Physical activity was computed as the average total metabolic equivalent-hours score per d on the basis of the frequency and duration of seven activities (walking, bicycling, standing, running, high-intensity activities, sleeping, and sedentary activity) over the preceding months^(^^26^^)^, which was categorised into quintiles. For only middle-aged women, occupation was considered (housewife, part-time job, or full-time job). Except for young women, education level was categorised as low, middle, and high (≤12, 13–15, and ≥16 years for middle-aged women and ≤9, 10–12, and ≥13 years for older women, respectively). Current marital status (yes or no) was also considered for middle-aged and older women.

Misreporting of EI was evaluated on the basis of the ratio of EI:BMR (the Goldberg's cut-off)^(^^27^^)^. Subjects were identified as plausible, under- and over-reporters of EI according to whether the individual's ratio was within, below or above the 95 % CI limits for agreement between EI:BMR and the respective physical activity level (PAL). In the present analysis, the PAL for sedentary lifestyle (i.e. 1·55)^(^^27^^)^ was applied for all subjects, because in all generations, time spent on sedentary activity (range of mean: 11·65–14·00 h/d) was predominant compared with other activities: walking (1·31–1·95 h/d), bicycling (0·14–0·31 h/d), standing (1·64–2·99 h/d), running (0·02–0·04 h/d), high-intensity activities (0·05–0·06 h/d) and sleeping (6·22–7·97 h/d). BMR was estimated according to an equation specifically developed for Japanese women, as follows: BMR (kJ/d) = (0·0481 × body weight (kg) + 0·0234 × body height (cm) − 0·0138 × age (years) − 0·9708)^(^^28^^,^^29^^)^. The 95 % confidence limits for agreement (upper and lower cut-off values) between EI:BMR and the PAL were calculated, taking into account CV in intakes and other components of energy balance (i.e. the within-subject variation in EI: 23 %; the precision of the estimated BMR relative to the measured BMR: 8·5 %; and the between-subject variation in PAL: 15 %)^(^^27^^)^. Consequently, under-reporters, plausible reporters and over-reporters were defined as having EI:BMR<1·09, 1·09–2·21 and >2·21, respectively.

### Statistical analysis

All statistical analyses were performed using SAS statistical software, version 9.3 (SAS Institute Inc.). All reported *P* values are two-tailed, and *P* < 0·05 was considered statistically significant. We decided *a priori* to conduct analyses for young, middle-aged and older women separately, mainly due to the use of different dietary assessment methods (i.e. the DHQ for young and middle-aged women and the BDHQ for older women) as well as the differences in potential confounding factors that should be considered. Descriptive data are presented as means and standard deviations for continuous variables and numbers and percentages of subjects for categorical variables. Differences in Japanese diet scores across categories of selected characteristics were examined by ANOVA.

Using the PROC REG procedure, stepwise forward regression analyses were carried out to investigate the contribution of the selected eighteen food groups for young and middle-aged women and fifteen food groups for older women to the inter-individual variation in Japanese diet scores. For those food groups contributing at least 1 % variation, multiple regression analyses were performed (using the PROC REG procedure) with predictive food groups as explanatory variables and Japanese diet scores as the response variable. We calculated regression CV of Japanese diet scores with a 1-sd increase of intake of each food group.

Multiple regression analyses were performed to explore the association of Japanese diet scores with nutrient intakes. The nutrients examined in the present study included protein, total, saturated, monounsaturated and polyunsaturated fats, carbohydrate, alcohol, dietary fibre, cholesterol, Na, K, Ca, Mg, Fe, vitamins A, E and C, and folate. These were selected mainly for assessing dietary intakes comprehensively while considering the current dietary intake patterns in Japanese women^(^^6^^,^^16^^,^^17^^)^. Using the PROC REG procedure, we calculated the adjusted regression coefficients (with se) of variation of intakes of selected nutrients by a five-point increase of Japanese diet scores. Potential confounding factors considered included survey year, residential block, size of residential area, weight status, current smoking, current alcohol drinking, dietary supplement use, medication use, physical activity and dietary reporting status. Further adjustment was made for living status and eating out in young women; eating out, occupation, education, current marital status and age (<45, 45–49, or ≥50 years) in middle-aged women; and living status, education, current marital status and age (61–69, 70–74, 75–79, or ≥80 years) in older women. The Japanese diet scores were analysed continuously after confirming the linearity of relationships using quintile categories. These analyses were repeated after excluding under- and over- reporters.

## Results

All of the young women were 18 years old while mean age was 47·7 (sd 3·9) years (range of age: 34–60 years) for middle-aged women and 74·4 (sd 5·2) years (range of age: 61–94 years) for older women (Table 1). Mean EI:BMR was 1·47 (sd 0·50) in young women, 1·66 (sd 0·49) in middle-aged women and 1·86 (sd 0·61) in older women.

**Table 1.** Basic and dietary characteristics of young, middle-aged and older Japanese women (Mean values and standard deviations)

	Young (*n* 3963)		Middle-aged (*n* 3800)		Older (*n* 2211)	
	Mean	sd	Mean	sd	Mean	sd
Age (years)	18	0	47·7	3·9	74·4	5·2
Body height (cm)	157·7	5·3	157·3	5·1	150·4	5·5
Body weight (kg)	52·0	7·8	54·5	8·2	51·6	7·9
BMI (kg/m^2^)	20·9	2·8	22·0	3·1	22·8	3·2
EI (kJ/d)	7242	2399	7717	2231	7362	2275
EI:BMR	1·47	0·50	1·66	0·49	1·86	0·61
Food intake (g/4184 kJ)						
White rice	151·0	79·9	134·8	72·1	165·1	69·7
Other grains*	17·0	49·6	15·7	46·0	–	–
Noodles	35·0	33·6	32·9	28·9	30·4	23·9
Bread	18·3	16·6	19·0	16·3	21·0	16·3
Other grain products*	6·7	8·3	6·2	8·1	–	–
Pulses	22·9	29·0	28·3	27·7	46·9	25·7
Nuts*	0·7	1·6	1·1	2·3	–	–
Potatoes	15·4	11·7	16·4	12·4	32·8	24·2
Sugar and confectioneries	54·3	27·2	47·7	22·6	41·2	25·2
Fruit	38·2	43·9	36·9	35·5	62·8	43·4
Total vegetables	124·3	79·9	133·9	70·3	181·8	81·6
Alcoholic beverages	1·2	15·0	48·5	111·7	9·8	46·1
Soft drinks	50·8	89·3	28·8	62·9	12·8	52·1
Fish and shellfish	25·4	16·2	30·7	16·1	58·9	31·3
Meat	36·8	19·3	36·4	17·9	32·1	18·0
Eggs	21·0	16·9	18·4	12·2	20·2	12·7
Dairy products	58·9	67·3	69·3	64·3	69·0	55·8
Seasonings	9·2	7·0	10·0	7·1	2·8	0·7
Nutrient intake						
Protein (% energy)	13·0	2·2	13·7	2·0	16·9	3·2
Total fat (% energy)	29·4	6·3	29·1	5·9	25·7	5·1
Saturated fat (% energy)	8·3	2·3	8·0	2·0	6·7	1·6
Monounsaturated fat (% energy)	10·4	2·7	10·4	2·5	8·9	2·0
Polyunsaturated fat (% energy)	6·5	1·6	6·7	1·5	6·4	1·4
Carbohydrate (% energy)	56·3	7·3	54·2	7·1	55·9	7·4
Alcohol (% energy)	0·1	0·7	1·8	4·1	0·5	2·3
Dietary fibre (g/4184 kJ)	6·2	2·0	6·7	2·0	8·0	2·2
Cholesterol (mg/4184 kJ)	171	75	165	57	231	76
Na (mg/4184 kJ)	2134	639	2279	618	2558	526
K (mg/4184 kJ)	1030	280	1194	276	1631	399
Ca (mg/4184 kJ)	246	94	267	93	367	112
Mg (mg/4184 kJ)	113	28	131	27	159	33
Fe (mg/4184 kJ)	3·6	0·9	3·7	0·9	5·1	1·2
Vitamin A (μg/4184 kJ)^†^	271	206	288	185	476	258
Vitamin E (mg/4184 kJ)^‡^	4·1	1·2	4·2	1·1	4·5	1·0
Folate (μg/4184 kJ)	147	52	156	48	239	75
Vitamin C (mg/4184 kJ)	46·5	23·5	47·9	23·2	85·5	31·2

EI, energy intake.

* Not available for older women.

† Retinol equivalents.

‡ α-Tocopherol.

Mean value of the original Japanese diet score was 33·6 (sd 7·5), 34·1 (sd 7·5) and 34·3 (sd 7·5) for young, middle-aged and older women, respectively (Table 2). The corresponding value of the modified Japanese diet score was 43·6 (sd 8·0), 44·1 (sd 8·2) and 50·4 (sd 8·0) for young, middle-aged and older women, respectively. The Pearson's correlation coefficient between the original and modified scores was 0·80, 0·81 and 0·76 for young, middle-aged and older women, respectively (all *P* < 0·0001). For the original Japanese diet score, the percentage of subjects with the maximum score for each category was low in all generations (0–37·1 %). In particular, none of the subjects reached the maximum score in the milk and fruits categories. For the modified score, the percentage of the subjects with the maximum score ranged from 0 to 97·4 %. Of note, almost all subjects reached the maximum score in the category of the fish and meat dish categories (83·9–97·4 %). For both scores, the Pearson's correlation coefficients between the score of each category and the total score were low to moderate in all generations (0·11–0·65), although the correlations for grain, vegetable, and fish and meat dishes were weaker in the modified score than in the original score.

**Table 2.** Descriptive statistics of the Japanese diet score in young, middle-aged and older Japanese women* (Mean values and standard deviations; percentages; Pearson's correlation coefficients)

			Original score^†^				Modified score^‡^			
	Intake^§^									
	Mean	sd	Mean	sd	% of subjects with maximum score	Pearson correlation with the total score^‖^	Mean	sd	% of subjects with maximum score	Pearson correlation with the total score^‖^
Young (*n* 3963)										
Grain dishes	3·8	1·1	8·3	1·7	26·0	0·42	8·5	1·8	40·1	0·25
Vegetable dishes	3·6	2·2	6·1	2·5	7·7	0·45	6·5	2·6	18·5	0·34
Fish and meat dishes	4·7	1·8	7·1	2·9	20·7	0·29	9·6	1·1	83·9	0·16
Milk	1·3	1·4	4·0	3·0	0	0·50	5·1	3·6	23·0	0·55
Fruits	0·7	0·8	2·9	2·5	0	0·38	3·2	2·7	4·6	0·39
Snacks and alcoholic beverages	316	164	5·1	4·1	24·7	0·57	5·1	4·1	24·7	0·62
Seasonings	2075	983	–	–	–	–	5·5	3·7	21·6	0·25
Total^¶^	–	–	33·6	7·5	0	–	43·6	8·0	0	–
Middle-aged (*n* 3800)										
Grain dishes	3·5	1·0	8·1	1·7	19·0	0·44	8·2	1·8	25·5	0·28
Vegetable dishes	3·9	1·9	6·7	2·4	10·1	0·38	7·0	2·4	22·5	0·31
Fish and meat dishes	5·1	1·6	6·7	2·9	16·5	0·24	9·8	0·8	91·6	0·11
Milk	1·5	1·4	4·5	3·2	0	0·49	5·9	3·6	28·7	0·56
Fruits	0·7	0·6	3·1	2·5	0	0·47	3·2	2·7	4·0	0·48
Snacks and alcoholic beverages	315	152	5·1	4·0	23·2	0·60	5·1	4·0	23·2	0·65
Seasonings	2200	941	–	–	–	–	4·9	3·6	14·3	0·27
Total^¶^	–	–	34·1	7·5	0	–	44·1	8·2	0	–
Older (*n* 2211)										
Grain dishes	3·5	1·1	8·1	1·9	22·9	0·52	8·2	1·9	32·6	0·28
Vegetable dishes	6·1	2·6	7·4	2·6	16·5	0·32	9·0	1·7	62·2	0·28
Fish and meat dishes	7·6	2·7	3·0	3·4	4·9	0·33	9·9	0·5	97·4	0·19
Milk	1·5	1·2	4·8	3·4	0	0·49	6·0	3·7	26·8	0·59
Fruits	1·1	0·8	4·9	2·8	0	0·35	5·3	3·1	13·6	0·49
Snacks and alcoholic beverages	276	164	6·0	4·1	37·1	0·49	6·0	4·1	37·1	0·58
Seasonings	1941	522	–	–	–	–	6·1	2·9	10·0	0·29
Total^¶^	–	–	34·3	7·5	0	–	50·5	8·0	0	–

* Dietary variables were energy adjusted using the density method to obtain the values per 7531 kJ of energy to enable comparisons with the recommended values.

† Recommended values (for the maximum score of 10) are as follows: grain dishes, 4–5 servings/7531 kJ; vegetable dishes, 5–6 servings/7531 kJ; fish and meat dishes, 3–4 servings/7531 kJ; milk, 2 servings/7531 kJ; fruits, 2 servings/7531 kJ; snacks and alcoholic beverages, <837 kJ of energy/7531 kJ. See Supplementary Table S1 for more details.

‡ Recommended values (for the maximum score of 10) are as follows: grain dishes, ≥4 servings/7531 kJ; vegetable dishes, ≥5 servings/7531 kJ; fish and meat dishes, ≥3 servings/7531 kJ; milk, ≥2 servings/7531 kJ; fruits, ≥2 servings/7531 kJ ; snacks and alcoholic beverages, <837 kJ of energy/7531 kJ; and seasoning, <1389 mg of Na/7531 kJ. See Supplementary Table S1 for more details.

§ Expressed as dietary serving per 7531 kJ of energy, except for snacks and alcoholic beverages (energy from snacks and alcoholic beverages (in kJ) per 7531 kJ of energy) and seasonings (Na from seasonings (in mg) per 7531 kJ of energy).

‖ All correlations were significant (*P* < 0·0001).

¶ Possible score ranging from 0 to 60 for the original score and 0 to 70 for the modified score.

Each of the Japanese diet scores was considerably differentially associated with selected characteristics in the three generations (Supplementary Table S2). However, living status, smoking, alcohol drinking and weight status showed similar associations in all generations. Individuals living alone, non-smokers and non-alcohol drinkers had higher mean scores, with there being no association for weight status.

Table 3 shows the food groups contributing (≥1 %) to the inter-individual variation in the Japanese diet scores. In total, these food groups accounted for 36 and 59 % (young women), 46 and 69 % (middle-aged women) and 47 and 80 % (older women) of variation of the original and modified diet scores, respectively. In young women, sugar and confectioneries, meat, soft drinks, and fish and shellfish were negatively associated with the original score, while fruit and total vegetables showed positive associations. In the case of the modified score, similar associations were observed (except for no associations for meat and fish and shellfish), but dairy products and seasonings emerged as positive and negative predictors, respectively. In middle-aged women, sugar and confectioneries, meat, soft drinks, fish and shellfish, and alcoholic beverages were negatively associated with the original score, but fruit was positively associated with the original score. However, application of the modified score revealed different associations (except for sugar and confectioneries, fruit, and alcoholic beverages), with positive associations with total vegetables, dairy products, and bread and a negative association with seasonings. For older women, fruit, dairy products, bread, white rice and noodles were positively associated with the original score. Similar associations were found for the modified score, except for negative associations with sugar and confectioneries, alcoholic beverages and seasoning (as well as no associations for bread, white rice and noodles).

**Table 3.** Food groups contributing to inter-individual variation in the Japanese diet score in young, middle-aged and older Japanese women* (Regression coefficients with their standard errors and partial determination coefficients)

	Young (*n* 3963)						Middle-aged (*n* 3800)						Older (*n* 2211)					
	Original score^†^			Modified score^‡^			Original score^†^			Modified score^‡^			Original score^†^			Modified score^‡^		
	*β*^§^	se	Partial *R*^2^	*β*^§^	se	Partial *R*^2^	*β*^§^	se	Partial *R*^2^	*β*^§^	se	Partial *R*^2^	*β*^§^	se	Partial *R*^2^	*β*^§^	se	Partial *R*^2^
	Model *R*^2^ 0·36			Model *R*^2^ 0·59				Model *R*^2^ 0·46		Model *R*^2^ 0·69			Model *R*^2^ 0·47			Model *R*^2^ 0·80		
Sugar and confectioneries	−3·60	0·10	0·22	−3·63	0·08	0·26	−3·50	0·10	0·12	−3·68	0·08	0·13	–‖	–‖	–‖	−4·48	0·08	0·16
Meat	−1·54	0·10	0·05	–‖	–‖	–‖	−1·49	0·09	0·04	–‖	–‖	–‖	–‖	–‖	–‖	–‖	–‖	–‖
Soft drinks	−1·41	0·10	0·03	−1·27	0·09	0·02	−0·85	0·09	0·01	–‖	–‖	–‖	–‖	–‖	–‖	–‖	–‖	–‖
Fruit	1·22	0·10	0·03	2·72	0·09	0·09	2·39	0·09	0·15	2·76	0·08	0·15	2·78	0·12	0·15	3·08	0·08	0·17
Total vegetables	1·01	0·10	0·01	1·47	0·09	0·04	–‖	–‖	–‖	1·19	0·08	0·02	–‖	–‖	–‖	–‖	–‖	–‖
Fish and shellfish	−0·79	0·10	0·01	–‖	–‖	–‖	−1·14	0·10	0·02	–‖	–‖	–‖	–‖	–‖	–‖	–‖	–‖	–‖
Dairy products	–‖	–‖	–‖	2·99	0·09	0·14	–‖	–‖	–‖	2·92	0·08	0·23	2·16	0·12	0·07	2·90	0·08	0·27
Seasonings	–‖	–‖	–‖	−0·61	0·04	0·03	–‖	–‖	–‖	−2·04	0·08	0·05	–‖	–‖	–‖	−3·37	0·08	0·16
Alcoholic beverages	–‖	–‖	–‖	–‖	–‖	–‖	−2·86	0·10	0·12	−2·73	0·08	0·10	–‖	–‖‖	–‖	−1·48	0·08	0·03
Bread	–‖	–‖	–‖	–‖	–‖	–‖	–‖	–‖	–‖	0·41	0·08	0·01	1·92	0·12	0·06	–‖	–‖	–‖
White rice	–‖	–‖	–‖	–‖	–‖	–‖	–‖	–‖	–‖	–‖	–‖	–‖	4·73	0·13	0·15	–‖	–‖	–‖
Noodles	–‖	–‖	–‖	–‖	–‖	–‖	–‖	–‖	–‖	–‖	–‖	–‖	1·42	0·12	0·03	–‖	–‖	–‖

*β*, Regression coefficient.

* Food groups listed are those contributing at least 1 % of the variation of the diet score based on the stepwise forward regression analysis with eighteen food groups (white rice; other grains; noodles; bread; other grain products; pulses; nuts; potatoes; sugar and confectioneries; fruits; total vegetables; alcoholic beverages; soft drinks; fish and shellfish; meat; eggs; dairy products; and seasonings) in young and middle-aged women and fifteen food groups (white rice; noodles; bread; pulses; potatoes; sugar and confectioneries; fruit; total vegetables; alcoholic beverages; soft drinks; fish and shellfish; meat; eggs; dairy products; and seasonings) in older women as explanatory variables and the diet score as the response variable.

† Recommended values (for the maximum score of 10) are as follows: grain dishes, 4–5 servings/7531 kJ; vegetable dishes, 5–6 servings/7531 kJ; fish and meat dishes, 3–4 servings/7531 kJ; milk, 2 servings/7531 kJ; fruits, 2 servings/7531 kJ; snacks and alcoholic beverages, <837 kJ of energy/7531 kJ. See Supplementary Table S1 for more details.

‡ Recommended values (for the maximum score of 10) are as follows: grain dishes, ≥4 servings/7531 kJ; vegetable dishes, ≥5 servings/7531 kJ; fish and meat dishes, ≥3 servings/7531 kJ; milk, ≥2 servings/7531 kJ; fruits, ≥2 servings/7531 kJ; snacks and alcoholic beverages, <837 kJ of energy/7531 kJ; seasoning, <1389 mg of Na/7531 kJ. See Supplementary Table S1 for more details.

§ Models with listed variables as the explanatory variables and the diet score as the response variable; regression coefficients mean the change of diet score with a 1-sd increase of intake of each food group (g/4184 kJ).

‖ Not contributing at least 1 % of the variation of diet score.

Associations of the Japanese diet scores with nutrient intakes are shown in Table 4. After adjustment for potential confounding factors, in young and middle-aged women, the original score was associated positively with intakes of protein (only young women), carbohydrate, Na, K, Ca, Mg, Fe, vitamins A, C and E, and folate and inversely with intakes of total, saturated, monounsaturated and polyunsaturated fats, cholesterol and alcohol. Similar associations were found for the modified score except for Na intake, which showed an inverse association. In older women, the original score was negatively associated with intakes of all nutrients (except for a positive association with carbohydrate and no association with vitamin C). However, the modified score was positively associated with intakes of protein, carbohydrate, dietary fibre, K, Ca, Mg, Fe, vitamins A, C and E, and folate and inversely with intakes of total, saturated, monounsaturated and polyunsaturated fats, cholesterol, alcohol and Na.

**Table 4.** Associations of the Japanese diet score with nutrient intakes in young, middle-aged and older Japanese women* (Regression coefficients with their standard errors)

	Young (*n* 3963)						Middle-aged (*n* 3800)						Older (*n* 2211)					
	Original score^†^			Modified score^‡^			Original score^†^			Modified score^‡^			Original score^†^			Modified score^‡^		
	*β*^§^	se	*P*	*β*^§^	se	*P*	*β*^§^	se	*P*	*β*^§^	se	*P*	*β*^§^	se	*P*	*β*^§^	se	*P*
Protein (% energy)	0·06	0·02	<0·0001	0·32	0·02	<0·0001	0·01	0·02	0·69	0·30	0·02	<0·0001	−0·47	0·05	<0·0001	0·15	0·04	0·001
Total fat (% energy)	−0·91	0·06	<0·0001	−0·75	0·06	<0·0001	−0·93	0·06	<0·0001	−0·58	0·06	<0·0001	−0·79	0·07	<0·0001	−0·40	0·07	<0·0001
Saturated fat (% energy)	−0·43	0·02	<0·0001	−0·25	0·02	<0·0001	−0·32	0·02	<0·0001	−0·09	0·02	<0·0001	−0·19	0·02	<0·0001	−0·07	0·02	0·001
Monounsaturated fat (% energy)	−0·32	0·03	<0·0001	−0·32	0·03	<0·0001	−0·38	0·03	<0·0001	−0·32	0·02	<0·0001	−0·32	0·03	<0·0001	−0·24	0·03	<0·0001
Polyunsaturated fat (% energy)	−0·04	0·02	0·02	−0·09	0·02	<0·0001	−0·11	0·02	<0·0001	−0·12	0·02	<0·0001	−0·16	0·02	<0·0001	−0·06	0·02	0·003
Carbohydrate (% energy)	0·89	0·08	<0·0001	0·51	0·07	<0·0001	1·63	0·07	<0·0001	0·95	0·07	<0·0001	1·46	0·10	<0·0001	0·63	0·10	<0·0001
Alcohol (% energy)	−0·019	0·007	0·006	−0·018	0·006	0·005	−0·66	0·04	<0·0001	−0·59	0·04	<0·0001	−0·24	0·03	<0·0001	−0·26	0·03	<0·0001
Dietary fibre (g/4184 kJ)	0·34	0·02	<0·0001	0·30	0·02	<0·0001	0·36	0·02	<0·0001	0·32	0·02	<0·0001	−0·06	0·03	0·05	0·42	0·03	<0·0001
Cholesterol (mg/4184 kJ)	−4·08	0·82	<0·0001	−1·77	0·76	0·02	−5·15	0·63	<0·0001	−2·18	0·58	0·0002	−13·55	1·06	<0·0001	−4·52	1·03	<0·0001
Na (mg/4184 kJ)	38·94	7·01	<0·0001	−70·81	6·36	<0·0001	32·10	7·01	<0·0001	−74·18	6·25	<0·0001	−67·57	7·36	<0·0001	−55·52	6·97	<0·0001
K (mg/4184 kJ)	33·59	2·97	<0·0001	63·55	2·58	<0·0001	33·99	3·04	<0·0001	60·09	2·62	<0·0001	−32·09	5·63	<0·0001	83·23	5·05	<0·0001
Ca (mg/4184 kJ)	8·55	1·02	<0·0001	22·13	0·87	<0·0001	9·97	1·02	<0·0001	24·72	0·84	<0·0001	−6·63	1·60	<0·0001	23·76	1·43	<0·0001
Mg (mg/4184 kJ)	3·35	0·30	<0·0001	5·05	0·26	<0·0001	2·81	0·30	<0·0001	4·44	0·27	<0·0001	−3·31	0·47	<0·0001	5·37	0·43	<0·0001
Fe (mg/4184 kJ)	0·03	0·01	0·001	0·05	0·01	0·02	0·06	0·01	<0·0001	0·07	0·01	<0·0001	−0·18	0·02	<0·0001	0·08	0·02	<0·0001
Vitamin A (μg/4184 kJ)^‖^	9·82	2·25	<0·0001	19·36	2·05	<0·0001	13·38	2·09	<0·0001	20·89	1·87	<0·0001	−21·44	3·73	<0·0001	14·19	3·53	<0·0001
Vitamin E (mg/4184 kJ)^¶^	0·04	0·01	0·0004	0·06	0·01	<0·0001	0·03	0·01	0·02	0·05	0·01	<0·0001	−0·14	0·01	<0·0001	0·05	0·01	<0·0001
Folate (μg/4184 kJ)	6·17	0·56	<0·0001	7·03	0·51	<0·0001	6·29	0·53	<0·0001	7·11	0·48	<0·0001	−6·51	1·05	<0·0001	9·92	0·98	<0·0001
Vitamin C (mg/4184 kJ)	3·23	0·25	<0·0001	3·89	0·22	<0·0001	3·67	0·25	<0·0001	3·53	0·23	<0·0001	−0·57	0·44	0·24	6·55	0·39	<0·0001

*β*, Regression coefficient.

* Adjustment was made for survey year, residential block, size of residential area, weight status, current smoking, current alcohol drinking, dietary supplement use, medication use, physical activity and dietary reporting status. For young women, additional adjustment was made for living status and eating out. For middle-aged women, additional adjustment was made for eating out, occupation, education, current marital status and age. For older women, additional adjustment was made for living status, education, current marital status and age.

† Recommended values (for the maximum score of 10) are as follows: grain dishes, 4–5 servings/7531 kJ; vegetable dishes, 5–6 servings/7531 kJ; fish and meat dishes, 3–4 servings/7531 kJ; milk, 2 servings/7531 kJ; fruits, 2 servings/7531 kJ; snacks and alcoholic beverages, <837 kJ of energy/7531 kJ. See Supplementary Table S1 for more details.

‡ Recommended values (for the maximum score of 10) are as follows: grain dishes, ≥4 servings/7531 kJ; vegetable dishes, ≥5 servings/7531 kJ; fish and meat dishes, ≥3 servings/7531 kJ; milk, ≥2 servings/7531 kJ; fruits, ≥2 servings/7531 kJ; snacks and alcoholic beverages, <837 kJ of energy/7531 kJ; seasoning, <1389 mg of Na/7531 kJ. See Supplementary Table S1 for more details.

§ Regression coefficients mean the change of nutrient intakes with a five-point increase of the diet score.

‖ Retinol equivalents.

¶ α-Tocopherol.

## Discussion

To our knowledge, this is the first study to examine the food-based diet quality scores in relation to nutrient intakes in three generations of Japanese women. We found that the diet score on the basis of the Japanese food guide Spinning Top (the original score) was positively associated with favourable dietary intake patterns, including higher intakes of protein, carbohydrate, K, Ca, Mg, Fe, vitamins A, C and E, and folate and lower intakes of total, saturated, monounsaturated and polyunsaturated fats, cholesterol and alcohol in young and middle-aged women. However, the score was also positively associated with Na intake. Additionally, in older women, the original score was inversely associated with intake of almost all nutrients examined. These results suggest that the diet score based on the Japanese food guide Spinning Top may not adequately express the quality of the Japanese dietary pattern. On the other hand, the modified score was associated with diet quality only in the expected direction in all three generations, including higher intakes of protein, carbohydrate, K, Ca, Mg, Fe, vitamins A, C and E, and folate and lower intakes of total, saturated, monounsaturated and polyunsaturated fats, cholesterol, Na and alcohol. Thus, the modified score may be a useful tool for assessing the quality of Japanese diets.

To our knowledge, only two studies have assessed the adherence to the food-based Japanese dietary guidelines on the basis of the original scoring system (which is identical for the original score used here). In a prospective cohort study in 13 355 men and 15 724 women, higher adherence to the dietary guidelines was associated with lower risk of future mortality in women (but not in men)^(^^8^^)^. Another study in 1083 young Japanese women showed that higher adherence was associated with lower waist circumference and LDL-cholesterol concentrations^(^^9^^)^, although it was not associated with other metabolic risk factors, including BMI, systolic and diastolic blood pressure, total and HDL-cholesterol, TAG, glucose, glycated Hb and insulin concentrations. Nevertheless, higher adherence was associated not only with favourable dietary intake patterns (such as higher intakes of protein, carbohydrate, dietary fibre, K and vitamin C)^(^^8^^,^^9^^)^ but also with less favourable dietary intake patterns (such as higher intakes of total, saturated, monounsaturated and polyunsaturated fat^(^^8^^)^, cholesterol^(^^8^^)^ and Na^(^^8^^,^^9^^)^).

In the present study, the original scoring system was generally associated with favourable nutrient intake patterns in young and middle-aged women. Nevertheless, the original score was also positively associated with Na intake. For older women, different associations were found. For nutrients, only carbohydrate was positively associated with the original score, with all other nutrients showing inverse associations. For food groups, we found that major carbohydrate-rich food groups, such as white rice, bread, and noodles as well as fruits and dairy products, were positive predictors for the original score. This suggests that the original score mainly reflects carbohydrate in older women. For many subjects (especially in the older generation) in this study, intakes of grains, vegetables, fish and meat, milk and fruits were higher than the upper cut-off value for the maximum score. In particular, almost all middle-aged and older women had intakes of fish and meat dishes above the upper cut-off values. This may explain why all nutrients except for carbohydrate were inversely associated with the original score in older women. Moreover, a higher intake of Na may be due to higher intakes of vegetables and meat and fish because in Japan these foods are usually accompanied by seasonings of salty taste, such as salt, soya sauce and miso. This highlights why seasonings need to be taken into consideration and why the original scoring system may not be a useful tool to evaluate the quality of the Japanese diet.

Na intakes have been shown to be much higher than the WHO recommendation in the Japanese population^(^^12^^)^. By using 4-d diet records and two 24-h urinary excretions, mean intake of Na was 4000 mg/d (*n* 392)^(^^13^^)^ and 5506 mg/d (*n* 760)^(^^12^^)^, respectively, in Japanese adults aged 20–69 years. The greatest contributor was seasonings such as soya sauces, miso, salt and soup stock, which accounted for 61·7 % of total Na intake^(^^13^^)^. Thus, we considered Na intake from seasonings when developing the modified score. Moreover, the modified score was calculated without applying the upper cut-off values for those dietary components where higher intakes are advocated, namely grain dishes, vegetable dishes, fish and meat dishes, milk and fruit. This decision was because it is difficult or uncertain in determining the amount by which such components should increase, and is in line with other scoring systems, such as Healthy Eating Index-2010^(^^18^^)^. As expected, the score of subjects with high intakes of grain dishes, vegetable dishes, fish and meat dishes, milk and fruit increased in all generations (particularly in the older). Consequently, the modified score was positively associated only with favourable nutrient intake patterns across all generations studied.

The major strength of the present study is a comprehensive investigation on the associations between the food-based diet quality scores and nutrient intakes in three generations of women who lived over a wide geographical range of Japan and had a variety of dietary and lifestyle patterns. However, there are also several limitations. First, given the proportion of the current Japanese adolescents who study in college or university (57 %)^(^^30^^)^, our participants (i.e. dietetic students and their mothers and grandmothers or acquaintances) are likely to have relatively high socio-economic statuses. Further, dietetic students may be more conscious of their diet and have eating disorders compared with the general population, although the present survey was carried out, in most institutions, within 1 month after the dietetic course began in order to minimise the influence of dietetic education. Additionally, the response rate was not high enough, especially in the grandmother generation (33·2 %), which may cause self-selection bias. Thus, our results might not be applicable to the general Japanese population.

Second, because different dietary assessment questionnaires were used for young and middle-aged (DHQ) and older (BDHQ) women, it is not possible to directly compare dietary intakes (or diet quality scores) across the three age categories. Nevertheless, we found that the modified score was similarly associated with nutrient intakes in all generations. Additionally, although the DHQ and the BDHQ we used have shown satisfactory relative validity for a wide range of nutrients and foods^(^^20^^,^^21^^)^, all dietary assessment methods suffer from measurement error. Moreover, our DHQ and BDHQ were not designed specifically to measure adherence to the food-based Japanese guidelines. Actually, while dietary guidelines are available in many countries, there are unfortunately few tools specifically designed for assessing dietary adherence. In such a situation, assumptions have to be made, which may be different among researchers, possibly producing different results. Thus it may be important to develop specific tools that could be used in epidemiological studies.

Third, each category of the diet score had the same weight, that is, these contribute equally to the total score. However, it is not plausible that all categories have the same health impact^(^^31^^)^. Therefore, it seems more appropriate to ascribe greater weights to those items that affect health status to a greater extent. In most indexes such as the Mediterranean diet score^(^^32^^)^ and Healthy Diet Indicator^(^^33^^)^, all individual categories have the same weight. Conversely, the Diet Quality Index-International, for example, has attributed different weights to different items^(^^34^^)^, although it is not clear how their scores for each categories were derived. In any case, it is very difficult to substantiate choices for different weights of the score items. Actually, how to weight the relative contribution of indicators to total scores is currently unknown with little guidance available^(^^31^^)^.

In conclusion, this cross-sectional study showed that the modified diet score seems to be a useful tool to assess diet quality in young, middle-aged and older Japanese women. Further research is needed to validate the score in relation to biochemical and clinical indicators of nutritional status. Also, utility of the score needs to be examined in other populations such as men and children.

## Supplementary material

For supplementary material accompanying this paper visit http://dx.doi.org/10.1017/jns.2016.36. click here to view supplementary material
