# Supplementary material for: Development of a food-based diet quality score for Japanese: associations of the score with nutrient intakes in young, middle-aged and older Japanese women
Source: J Nutr Sci. 2016 Oct 28;5:e41. doi: 10.1017/jns.2016.36 (PMC5465807; doi:10.1017/jns.2016.36)
Supplement: Supplementary file 1 [file S2048679016000367sup001.pdf]

Supplemental Table 1. Scoring system of the Japanese diet score used in the present study

Category*	Food groups	Food item		Original score		Modified score	
		DHQ†	BDHQ‡	Serving consumed§	Score given‖	Serving consumed§	Score given
Grain dishes	White rice	White rice (1 item)	White rice (1 item)	< 4	10 × (serving consumed)/4	< 4	10 × (serving consumed)/4
	Other grains	White rice mixed with barley; white rice with germ; half-milled rice; 70 %-milled rice; brown rice（5 items)	---¶	4 to 5	10	≥ 4	10
				> 5	10 - 10 × [(serving consumed) - 5]/5		
	Noodles	Japanese noodles (buckwheat, instant noodles and Japanese wheat noodles); instant noodles; Chinese noodles; spaghetti and macaroni (4 items)	Buckwheat and instant noodles; Japanese wheat noodles; instant noodles and Chinese noodles; spaghetti and macaroni (4 items)				
	Bread	White bread; butter roll; croissant (3 items)	Breads (including white bread and Japanese bread with a sweet filling) (1 item)				
	Other grain products	Pizza; Japanese-style pancakes; cornflakes (3 items)	---¶				
Vegetable dishes	Potatoes	French fries; potatoes; sweet potatoes, yams, and taro; konnyaku (i.e., devil's tongue jelly) (4 items)	Potatoes (all varieties) (1 item)	< 5	10 × (serving consumed)/5	< 5	10 × (serving consumed)/5
				5 to 6	10	≥ 5	10
				> 6	10 - 10 × [(serving consumed) - 6]/6		
	Nuts	Peanuts; other nuts (2 items)	---¶				
	Total vegetable	Carrots; pumpkins; tomatoes; green peppers; broccoli; green leafy vegetables; salted pickled plums; cabbage; cucumbers; lettuce; Chinese cabbage; bean sprouts; radishes; onions; cauliflower; eggplants; burdock; lotus root; salted pickles (leafy vegetables); salted pickles (others); mushrooms; wakame and hijiki seaweed; laver (i.e., dried, edible seaweed); vegetable juice; tomato juice (25 items)	Salted green and yellow vegetable pickles; other salted vegetables (excluding salted pickled plums); raw vegetables used in salad (e.g., cabbage and lettuce); green leafy vegetables including broccoli; cabbage and Chinese cabbage; carrots and pumpkins; radishes and turnips; other root vegetables (e.g., onions, burdock, and lotus root); tomatoes, tomato ketchup, boiled tomato, and stewed tomato; mushrooms (all varieties); seaweeds (all varieties); vegetable juice (12 items)				

Supplemental Table 1. Continued

Category*	Food groups	Food item: DHQ†	Food item: BDHQ‡	Original score: Serving consumed§	Original score: Score given||	Modified score: Serving consumed§	Modified score: Score given
Fish and meat dishes	Meat	Ground beef and pork; chicken; pork; beef; liver; ham and sausages; bacon (7 items)	Chicken (including ground chicken); pork and beef (including ground and beef); ham, sausages, and bacon; liver (4 items)	< 3<br>3 to 4<br>> 4	10 × (serving consumed)/3<br>10<br>10 - 10 × [(serving consumed) - 4]/4	< 3<br>≥ 3	10 × (serving consumed)/3<br>10
	Egg	Eggs (1 item)	Eggs (1 item)				
	Pulses	Tofu (i.e., soybean curd); tofu products; natto (i.e., fermented soybeans); boiled beans; soy milk (5 items)	Tofu (i.e., soybean curd) and tofu products; natto (i.e., fermented soybeans) (2 item)				
	Fish and selfish	Dried fish; small fish with bones; canned tuna; eel; white meat fish; red meat fish; oily fish; ground fish meat products; shrimp and crab; squid and octopus; oysters; other shellfish; fish eggs; boiled fish and shellfish in soy sauce; salted fish intestines (15 items)	Squid, octopus, shrimp, and clam; small with bones; canned tuna; dried fish and salted fish (including salted mackerel, salted salmon, and dried horse mackerel); oily fish (including sardines, mackerel, saury, amberjack, herring, eel, and fatty tuna); non-oily fish (including salmon, trout, white meat fish, freshwater fish, and bonito) (6 items)				
Milk	Dairy products	Sweetened yogurt; non-sweetened yogurt; moderately sweetened yogurt; cheese; cottage cheese; low-fat milk; full-fat milk; cream or creamer added to coffee (8 items)	Low-fat milk and yogurt; full-fat milk and yogurt (2 items)	< 2<br>2<br>> 2	10 × (serving consumed)/2<br>10<br>10 - 10 × [(serving consumed) - 2]/2	< 2<br>≥ 2	10 × (serving consumed)/2<br>10
Fruits	Fruit	Raisins; canned fruits; oranges; bananas; apples; strawberries; grapes; peaches; pears; persimmons; kiwi fruits; melons; watermelons; fruit juice (14 items)	Citrus fruit; strawberries, persimmons, and kiwi fruits; other fruit (3 items)	< 2<br>2<br>> 2	10 × (serving consumed)/2<br>10<br>10 - 10 × [(serving consumed) - 2]/2	< 2<br>≥ 2	10 × (serving consumed)/2<br>10
Snacks and alcoholic beverages	Alcoholic beverages	Beer; Sake; Shochu; Shochu mixed with water or a carbonated beverage; whiskey; wine; (6 items)	Beer; Sake; Shochu; whiskey; wine; (5 items)	≤ 837 kJ<br>> 837 kJ	10<br>10 - 10 × [(energy consumed in kJ) - 837]/837	≤ 837 kJ<br>> 837 kJ	10<br>10 - 10 × [(energy consumed in kJ) - 837]/837||

Supplemental Table 1. Continued

Category*	Food groups	Food item: DHQ†	Food item: BDHQ‡	Original score: Serving consumed§	Original score: Score given‖	Modified score: Serving consumed§	Modified score: Score given
	Sugar and confectioneries	Jam and marmalade; sugar for coffee and black tea; Japanese bread with a sweet filling; pancakes; potato chips; rice crackers; snacks made from wheat flour; Japanese sweets with azuki beans; Japanese sweets without azuki beans; cakes; cookies and biscuits; chocolates; candies, caramels, and chewing gum; jellies; doughnuts; ice cream (premium); ice cream (unspecified varieties); ice cream (regular) (18 items)	Cakes, cookies, and biscuits; Japanese sweets; rice crackers, rice cakes, and Japanese-style pancakes; ice cream; sugar for coffee and black tea (5 items)				
	Soft drinks	Fruit juice excluding 100 % juice; cocoa; lactic acid bacteria beverages; sugar-sweetened soft drinks; nutritional supplement drinks (5 items)	Cola and sugar-sweetened soft drinks (1 item)				
Seasonings	Seasonings	Ketchup; fat-free salad dressing; table salt; salt used during cooking; soy sauce; curry and roux in stew; miso as seasoning (7 items)	Table salt; soy sauce (2 items)	---	---	≤1389 mg** (sodium)	10
						>1389 mg** (sodium)	10-10×((sodium consumed in mg)-1389**)/ 1389‖

DHQ, diet history questionnaire; BDHQ, brief diet history questionnaire.

* By definition, one serving of grain dishes contained 40 g carbohydrate; one serving of vegetable dishes was 70 g; one serving of fish and meat dishes contained 6 g protein; one serving of milk contained 100 mg calcium; and one serving of fruits was 100 g.

† Used for young and middle-aged women. The following food groups were not categorized and thus not used in the calculation described here: tea and coffee (3 items), sugar-free soft drinks (1 item), fats and oils (5 items), sugar used during cooking (1 item), soups (5 items), nutritional supplement bars (1 item), artificial sweeteners (1 item), and drinking water (1 item).

‡ Used for older women. The following food groups were not categorized and thus not used in the calculation described here: tea and coffee (3 items), fats and oils (2 items), sugar used during cooking (1 item), and soups (2 items).

§ Per 7531 kJ of energy.

‖ When the calculation produced a negative score because of excess servings, energy, or sodium the score was converted to 0.

¶ Not available in BDHQ.

** This value (1389 mg of sodium) was based on the 10th percentile of energy-adjusted sodium intake from seasonings (per 7531 kJ) in older women.

Supplemental Table 2. The Japanese diet score according to categories of selected characteristics in young, middle-aged, and older Japanese women (Numbers and percentages; mean values and standard deviations)

	Young (*n* 3963)								Middle-aged (*n* 3800)								Older (*n* 2211)							
			Original score*			Modified score†					Original score*			Modified score†					Original score*			Modified score†		
	n	%	Mean	SD	*P*	Mean	SD	*P*	*n*	%	Mean	SD	*P*	Mean	SD	*P*	*n*	%	Mean	SD	*P*	Mean	SD	*P*
Survey year					<0.0001			<0.0001					<0.0001			<0.0001					<0.0001			0.003
2011	2461	62.1	33.0	7.3		43.0	7.8		2311	60.8	35.2	7.4		43.6	8.2		1408	63.7	34.9	7.4		50.9	8.0	
2012	1502	37.9	34.6	7.6		44.5	8.3		1489	39.2	33.3	7.6		44.8	8.3		803	36.3	33.1	7.6		49.8	8.0	
Residential block					<0.0001			<0.0001					0.0004			0.007					<0.0001			0.04
Hokkaido and Tohoku	379	9.6	33.5	7.6		43.5	8.1		383	10.1	34.4	7.4		44.7	8.4		209	9.5	34.2	6.9		50.1	8.0	
Kanto	1144	28.9	34.5	7.5		44.3	8.3		1074	28.3	34.7	7.3		44.7	8.3		547	24.7	32.8	7.8		50.0	8.1	
Hokuriku and Tokai	808	20.4	33.6	7.2		43.7	8.0		840	22.1	34.1	7.6		43.7	8.4		539	24.4	34.7	7.7		50.0	8.4	
Kinki	480	12.1	31.5	7.1		41.7	7.6		494	13.0	32.9	7.4		43.4	8.2		273	12.4	35.5	7.7		51.5	8.0	
Chugoku and Shikoku	557	14.1	33.6	7.4		43.4	8.1		520	13.7	33.7	7.6		43.6	7.9		359	16.2	34.7	7.4		51.1	7.8	
Kyushu	595	15.0	33.7	7.6		43.6	7.6		489	12.9	34.6	7.6		44.1	8.1		284	12.8	34.6	6.7		50.9	7.4	
Size of residential area					0.89			0.49					0.04			0.13					0.05			0.05
City with a population ≥ 1million	739	18.7	33.7	7.5		43.3	8.0		595	15.7	33.5	7.3		43.6	7.9		285	12.9	34.4	7.9		51.4	7.8	
City with a population < 1million	2914	73.5	33.6	7.5		43.6	8.1		2821	74.2	34.2	7.5		44.1	8.3		1682	76.1	34.1	7.5		50.3	8.1	
Town and village	310	7.8	33.5	7.2		44.0	7.6		384	10.1	34.6	7.4		44.7	8.0		244	11.0	35.4	7.0		51.0	7.7	
Living status					<0.0001			<0.0001					---			---					<0.0001			<0.0001
Living alone	923	23.3	35.1	7.7		44.8	8.3		---	---	---	---		---	---		355	16.1	35.9	7.4		52.9	7.6	
Living with family	2910	73.4	33.1	7.4		43.1	7.9		---	---	---	---		---	---		1856‡	83.9	34.0	7.5		50.0	8.0	
Living with others	130	3.3	34.7	6.8		44.7	7.8		---	---	---	---		---	---		---	---	---	---		---	---	
Weight status§					0.42			0.14					0.48			0.91					0.25			0.15
Underweight	685	17.3	33.3	7.3		43.1	8.1		320	8.4	33.9	7.5		44.0	8.3		167	7.6	33.4	7.1		50.2	8.7	
Nomal weight	3015	76.1	33.7	7.5		43.6	8.0		2904	76.4	34.2	7.5		44.1	8.3		1555	70.3	34.3	7.6		50.7	8.0	
Overweight	263	6.6	33.9	7.5		44.3	8.0		576	15.2	33.8	7.4		44.2	8.2		489	22.1	34.5	7.4		49.9	7.7	
Current smoking					0.02			0.06					<0.0001			<0.0001					0.001			<0.0001
Yes	7	0.18	30.8	6.5		38.0	5.4		290	7.6	30.2	8.1		39.7	8.7		59	2.7	31.1	8.1		45.2	8.7	
No	3956	99.8	37.2	7.5		43.6	8.0		3510	92.4	34.5	7.3		44.5	8.1		2152	97.3	34.4	7.5		50.6	8.0	

Supplemental Table 2. Continued

	Young (*n* 3963)								Middle-aged (*n* 3800)								Older (*n* 2211)							
			Original score*			Modified score†					Original score*			Modified score†					Original score*			Modified score†		
	n	%	Mean	SD	*P*	Mean	SD	*P*	*n*	%	Mean	SD	*P*	Mean	SD	*P*	*n*	%	Mean	SD	*P*	Mean	SD	*P*
Current alcohol dirinking					<0.0001			<0.0001					<0.0001			<0.0001					<0.0001			<0.0001
Yes	237	6.0	31.8	7.2		41.5	7.5		1872	49.3	33.1	7.6		42.9	8.3		440	19.9	33.0	7.8		48.8	8.6	
No	3726	94.0	33.7	7.5		43.7	8.0		1928	50.7	35.1	7.2		45.3	8.0		1712	80.1	34.6	7.4		50.9	7.8	
Dietary supplement use					0.92			0.32					0.07			0.001					0.04			<0.0001
Yes	257	6.5	33.6	7.2		44.0	7.6		982	25.8	34.5	7.4		44.8	8.1		664	30.0	34.8	7.5		51.6	7.6	
No	3706	93.5	33.6	7.5		43.5	8.1		2818	74.2	34.0	7.5		43.8	8.3		1547	70.0	34.1	7.5		50.0	8.2	
Medication use					0.10			0.002					0.02			0.64					0.003			0.0002
Yes	427	10.8	34.2	7.4		44.7	8.0		962	25.3	33.7	7.6		44.0	8.3		1700	76.9	34.7	7.4		50.8	7.9	
No	3536	89.2	33.5	7.5		43.4	8.0		2838	74.7	34.3	7.4		44.1	8.2		511	23.1	33.4	7.7		49.3	8.4	
Eating out					<0.0001			<0.0001					<0.0001			<0.0001					---			---
≤3 times/month	1685	42.5	32.3	7.9		44.4	8.1		2368	62.3	32.3	7.6		44.9	8.3		---	---	---	---		---	---	
Once per week	826	20.8	34.5	7.5		43.6	7.7		656	17.3	34.7	7.5		43.1	7.8		---	---	---	---		---	---	
2-3 times/week	790	19.9	33.6	7.2		42.7	7.8		445	11.7	33.6	7.1		42.9	8.2		---	---	---	---		---	---	
≥4 times/week	662	16.7	32.8	7.1		42.3	8.3		331	8.7	33.1	7.5		41.9	8.2		---	---	---	---		---	---	
Physical activity					0.39			0.95					0.09			0.28					0.75			0.01
Quartile 1 (lowest)	978	24.7	33.5	7.3		43.5	7.8		946	24.9	34	7.5		43.8	8.1		547	24.7	34.4	7.5		49.6	8.3	
Quartile 2	1017	25.7	33.9	7.3		43.6	7.9		950	25.0	34.5	7.6		44.3	8.3		558	25.2	34.5	7.5		50.4	7.9	
Quartile 3	1002	25.3	33.7	7.7		43.4	8.2		953	25.1	34.3	7.4		44.4	8.3		553	25.0	34.3	7.9		51.2	8.1	
Quartile 4	966	24.4	33.3	7.6		43.7	8.3		951	25.0	33.7	7.4		43.8	8.3		553	25.0	34.0	7.2		50.8	7.8	
Occupation					---			---					0.0001			<0.0001					---			---
Housewife	---	---	---	---		---	---		747	19.7	35.1	7.5		45.3	8.4		---	---	---	---		---	---	
Part-time job	---	---	---	---		---	---		1687	44.4	33.7	7.3		43.6	8.1		---	---	---	---		---	---	
Full-time job	---	---	---	---		---	---		1366	36.0	34.1	7.6		44.0	8.3		---	---	---	---		---	---	
Education					---			---					<0.0001			<0.0001					0.39			<0.0001
Low‖	---	---	---	---		---	---		1823	48.0	33.1	7.4		42.9	8.1		990	44.8	34.2	7.5		49.6	8.0	
Middle¶	---	---	---	---		---	---		1425	37.5	34.6	7.4		44.7	8.2		1005	45.5	34.5	7.4		51.0	8.0	
High**	---	---	---	---		---	---		552	14.5	36.2	7.2		46.6	7.9		216	9.8	33.8	8.1		52.4	7.8	

Supplemental Table 2. Continued

	Young (*n* 3963)								Middle-aged (*n* 3800)								Older (*n* 2211)							
			Original score*			Modified score†					Original score*			Modified score†					Original score*			Modified score†		
	n	%	Mean	SD	*P*	Mean	SD	*P*	*n*	%	Mean	SD	*P*	Mean	SD	*P*	*n*	%	Mean	SD	*P*	Mean	SD	*P*
Current marital status					---			---					0.001			0.002					0.003			0.19
Yes	---	---	---	---		---	---		3479	91.6	34.2	7.4		44.2	8.2		1346	60.9	33.9	7.4		50.3	7.9	
No	---	---	---	---		---	---		321	8.5	32.9	8.2		42.7	8.6		865	39.1	34.9	7.7		50.8	8.2	
Dietary reporting status††					<0.0001			<0.0001					<0.0001			<0.0001					<0.0001			<0.0001
Under-reporting	742	18.7	34.5	7.2		44.2	7.6		291	7.7	32.9	8.4		43.0	8.8		141	6.4	32.8	7.8		47.4	9.6	
Plausible reporting	2962	74.7	33.8	7.4		43.6	8.1		3085	81.2	34.6	7.4		44.4	8.2		1549	70.1	35.2	7.7		51.0	8.1	
Over-reporting	259	6.5	29.0	7.2		40.7	8.2		424	11.2	31.5	7.1		42.5	8.3		521	23.6	32.1	6.4		49.8	7.1	

† Possible score ranging from 0 to 70; see Supplemental Table 1 for more detail.

‡ Including women living with others because of the small numbers of subjects (*n* 4).

§ Underweight were defined as subjects with BMI <18.5 kg/m^2; normal weight defined as subjects with BMI ≥18.5 to <25 kg/m^2; overweight defined as subjects with BMI ≥25.0 kg/m^2.

|| ≤12 years for middle-aged women and ≤9 years for older women.

¶ 13-15 years for middle-aged women and 10-12 years for older women.

** ≥16 years for middle-aged women and ≥13 years for older women.

†† Under-reporting were defined as subjects with a ratio of reported energy intake to BMR (EI:BMR) <1.09; plausible reporting defined as subjects with EI:BMR 1.09-2.21; over-reporting defined as subjects with EI:BMR > 2.21.
